# Identification of a novel enhancer that binds Sp1 and contributes to induction of cold-inducible RNA-binding protein (cirp) expression in mammalian cells

**DOI:** 10.1186/1472-6750-12-72

**Published:** 2012-10-10

**Authors:** Yasuhiko Sumitomo, Hiroaki Higashitsuji, Hisako Higashitsuji, Yu Liu, Takanori Fujita, Toshiharu Sakurai, Marco M Candeias, Katsuhiko Itoh, Tsutomu Chiba, Jun Fujita

**Affiliations:** 1 Department of Clinical Molecular Biology, Graduate School of Medicine, Kyoto University, 54 Shogoin Kawaharacho, Sakyo-ku, Kyoto, 606-8507, Japan; 2 Department of Gastroenterology and Hepatology, Graduate School of Medicine, Kyoto University, Kyoto, 606–8507, Japan; 3 Current address: Department of Gastroenterology and Hepatology, Kobe City Medical, Center West Hospital, Kobe, 653-0013, Japan; 4 Department of Gastroenterology and Hepatology, Kinki University School of Medicine, Osaka, 589-8511, Japan; 5 Current address: Department of Bioimaging and Cell Signaling, Graduate School of Biostudies, Kyoto University, Kyoto, 606-8501, Japan

**Keywords:** Cold shock protein, Stress response, Enhancer, Transcription factor, Recombinant protein

## Abstract

**Background:**

There are a growing number of reports on the sub-physiological temperature culturing of mammalian cells for increased recombinant protein yields. However, the effect varies and the reasons for the enhancement are not fully elucidated. Expression of cold-inducible RNA-binding protein (*cirp*, also called *cirbp* or *hnRNP A18*) is known to be induced in response to mild, but not severe, hypothermia in mammalian cells. To clarify the molecular mechanism underlying the induction and to exploit this to improve the productivity of recombinant proteins, we tried to identify the regulatory sequence(s) in the 5′ flanking region of the mouse *cirp* gene.

**Results:**

By transiently transfecting HEK293 cells with plasmids expressing chloramphenicol acetyltransferase as a reporter, we found that the *cirp* 5′ flanking region octanucleotide 5′-TCCCCGCC-3′ is a mild-cold responsive element (MCRE). When 3 copies of MCRE were placed upstream of the CMV promoter and used in transient transfection, reporter gene expression was increased 3- to 7-fold at 32°C relative to 37°C in various cell lines including HEK293, U-2 OS, NIH/3T3, BALB/3T3 and CHO-K1 cells. In stable transfectants, MCRE also enhanced the reporter gene expression at 32°C, although more copy numbers of MCRE were necessary. Sp1 transcription factor bound to MCRE in vitro. Immunohistochemistry and chromatin immunoprecipitation assays demonstrated that more Sp1, but not Sp3, was localized in the nucleus to bind to the *cirp* regulatory region containing MCRE at 32°C than 37°C. Overexpression of Sp1 protein increased the expression of endogenous Cirp as well as a reporter gene driven by the 5′ flanking region of the *cirp* gene, and down-regulation of Sp1 had the opposite effect. Mutations within the MCRE sequence in the 5′ flanking region abolished the effects of Sp1 on the reporter gene expression both at 37°C and 32°C.

**Conclusions:**

Cold-induced, as well as constitutive, expression of *cirp* is dependent, at least partly, on MCRE and Sp1. The present novel enhancer permits conditional high-level gene expression at moderately low culture temperatures and could be utilized to increase the yield of recombinant proteins in mammalian cells.

## Background

Cold-inducible RNA-binding protein (Cirp, also called Cirbp or hnRNP A18) is the first cold shock protein identified in mammals [1,2]. Being composed of one consensus sequence-RNA binding domain and a carboxyl-terminal region containing several arginine glycine glycine motifs, it is structurally quite different from bacterial cold shock proteins. Expression of Cirp is induced in response to mild, but not severe, hypothermia. Cirp is also induced by cellular stresses such as UV irradiation and hypoxia [3,4]. Upon stress induction, Cirp shuttles from the nucleus to the cytoplasm, and affects stability and translation of its target mRNAs [5]. Cirp modulates cell cycle progression, protects cells from various stresses, and possibly functions as an oncoprotein [2,6-8]. Cirp is constitutively expressed in a variety of tissues and cells, predominantly in the testis and brain, and contributes to the maintenance of normal cellular functions as well. Recently, we have produced *cirp*-knockout mice and demonstrated that Cirp stimulates proliferation of undifferentiated spermatogonia by interacting with Dyrk1b/Mirk protein [9]. The mechanism(s) underlying the induction of cold shock proteins by mild hypothermia has not yet been fully elucidated.

Specificity protein 1 (Sp1) is a member of the large multigene family of Sp/Kruppel-like factor transcription factors that can activate or repress transcription in response to physiologic and pathologic stimuli [10]. These proteins share a highly conserved DNA binding domain, and through 3 adjacent C2H2-type zinc fingers at the C-terminus, they bind to GC boxes, CACCC boxes, and basic transcription elements. Sp1 through Sp4 form a subgroup that contains glutamine-rich transactivating domains. Sp1, Sp3, and Sp4 have 2 such domains, whereas Sp2 has only 1 and exhibits different DNA binding specificity. Sp1 and Sp3 are expressed ubiquitously, whereas Sp4 is expressed primarily in neural cells. Often in cooperation with other transcription factors, Sp proteins regulate the expression of numerous genes implicated in the control of a diverse array of cellular processes, such as cell growth, differentiation, apoptosis, and angiogenesis [11].

A number of biotechnology products currently marketed are large molecules, produced from genetically modified mammalian cell lines, and extracted through complex and lengthy purification procedures [12,13]. The expiration of patents for expensive protein drugs and the development of monoclonal antibodies as therapeutics have intensified competitive efforts to improve the productivity of recombinant proteins. One area in which there is currently a large volume of interest with regard to improving recombinant protein production in mammalian cells is through the use of lower temperature cultivation [12]. Mild hypothermic conditions result in prolonged generation time, maintenance of cell viability for longer periods, reduced glucose and glutamine consumption, suppressed release of waste products, delayed apoptosis, reduced protease activity and decreased O_2_ demand among others [1,6,14]. The overall effects, however, vary among cell lines, expression systems, and the product of interest, and opportunities exist for further enhancement of the cold shock effect on recombinant protein production in mammalian cells [12].

In the present study, we identified the mild-cold responsive element (MCRE) in the 5′ flanking region of the mouse *cirp* gene, which enhances gene expression at 32°C in cultured mammalian cells. We have found that Sp1 binds to the identified MCRE sequence, and that downregulation of Sp1 expression suppresses the induction of *cirp* gene expression at 32°C. We have also shown that MCRE can be utilized to increase the yield of recombinant proteins produced in mammalian cells.

## Results

### Identification of the *cis*-regulatory element that enhances gene expression at 32°C

**Figure 1:**
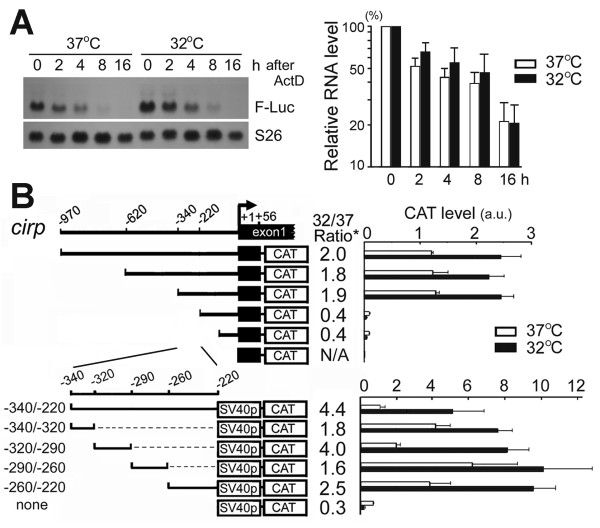
**Isolation of the *cirp* genomic fragments that increase expression at moderately low temperatures.** (**A**) Comparison of the degradation rates of reporter mRNA at 37°C and 32°C. NIH/3T3 Flp-In cells stably transfected with the pcDNA5/FRT vector expressing firefly luciferase (F-Luc) under the control of the *cirp −*970/+56 genomic region instead of the CMV promoter were transferred to 32°C or remained at 37°C for indicated times after starting incubation with 15 μg/ml actinomycin D (ActD). Expression of F-Luc was analyzed by northern blotting (20 μg of total RNA per lane), and compared with that of ribosomal protein S26 (left). Expression of F-Luc mRNA was quantified by quantitative RT-PCR, normalized to that of Tubb3 having a long mRNA half-life [27], and expressed as relative to time 0 (right). Values are mean ± SD, n = 3. Values do not significantly differ at 37°C and 32°C. (**B**) HEK293 cells were transiently co-transfected with CAT reporter constructs having the gene fragment upstream of the *cirp* transcription initiation site (+1) as indicated and plasmids expressing β-galactosidase (LacZ). The reporter constructs contained endogenous *cirp* promoter (upper panel) or SV40 minimal promoter (lower panel). Cells were maintained at 37°C or transferred to 32°C one day after transfection, and 36 hours later cell extracts were assayed. CAT protein level was normalized to LacZ activity. Bars represent the means ± SE of three to four determinations. a.u., arbitrary unit. *, Normalized CAT level at 32°C divided by that at 37°C. N/A, not applicable.

**Figure 2:**
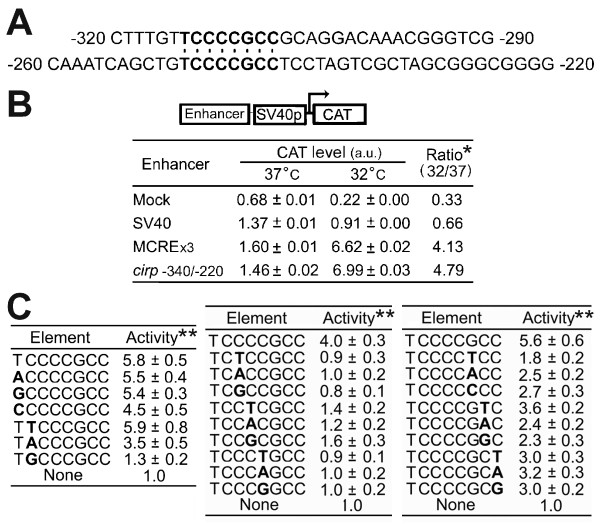
**Identification of mild-cold responsive element (MCRE) in the *cirp* gene.** (**A**) Sequence comparison of the *cirp* genomic fragments. Note the presence of common octanucleotide (bold). (**B**) Enhancer activity of MCRE. The indicated DNA fragments (Enhancer) were placed upstream of the SV40 promoter in pCAT promoter vector. HEK293 cells were co-transfected with the constructs and plasmids expressing β-galactosidase (LacZ). Cells were maintained at 37°C or transferred to 32°C on the following day, and 36 hours later cell extracts were assayed for CAT level and LacZ activity. Values are expressed as CAT protein level normalized to LacZ activity, and the means ± SE of three determinations are shown. a.u., arbitrary unit. *, CAT level normalized to LacZ at 32°C divided by that at 37°C. MCREx3, 3 copies of MCRE flanked on its 5′ and 3′ sides by T and G, respectively, and joined together. (**C**) Activities of mutant MCRE. Three copies of the indicated octanucleotide (Element) each with a mutation (bold) were assayed for the activity as in (**B**). **, normalized CAT level at 32°C divided by that at 37°C, and expressed as relative to the value obtained with pCAT promoter vector having no enhancer (none). The means ± SE of results from two independent transfections are shown.

To analyze the transcriptional regulation of *cirp* expression, we isolated an approximately 10 kb-long 5′ genomic fragment upstream of the transcription start site. When we inserted the gene fragment spanning from position −970 to +56 (+1 corresponds to the transcription start site) into the pcDNA5/FRT vector containing F-Luc cDNA without promoter and transfected NIH/3T3 Flp-In cells with this plasmid, the expression level of F-Luc mRNA was higher at 32°C than 37°C, whereas there was not much difference in the degradation rate of mRNA at 37°C and 32°C (Figure 1A). Therefore, we made a series of constructs containing different 5′ deletions of the −970/+56 fragment and tested their transcriptional activity by transiently transfecting HEK293 cells (Figure 1B). With the −970/+56 fragment, higher expression of the reporter was observed at 32°C than 37°C as expected. When the −340/-220 region was deleted from the −340/+56 fragment, the transcriptional activity dropped more than 10-fold and the activity at 32°C became less than that at 37°C. Negligible expression was observed without the −120/-1 fragment, suggesting the presence of basal promoter activity within it. In order to determine the enhancer fragment responsible for the response to moderate cold, we subdivided the −340/-220 fragment and tested each fragment for the enhancer activity using the SV40 minimal promoter. As shown in Figure 1B, the −320/-290 and −260/-220 fragments enhanced the expression more than twofold at 32°C relative to 37°C. We noticed that the octanucleotide sequence 5′-TCCCCGCC-3′ was common to both fragments (Figure 2A). When three copies of 5′-TTCCCCGCCG-3′ containing this octanucleotide were directly joined together and placed upstream of the SV40 promoter, the expression of CAT reporter was enhanced at 32°C as much as the *cirp −*340/-220 fragment (Figure 2B). Using the same expression constructs, we observed similar enhancing effects at 32°C in human U-2 OS cells, human HeLa cells, mouse NIH/3T3 cells, mouse BALB/3T3 cells and Chinese hamster CHO-K1 cells as well. Thus, we named this octanucleotide sequence as the mild-cold responsive element (MCRE). The mutants of MCRE with a replacement of the first base T by A, G, or C showed similar enhancer activities to that of the wild type (Figure 2C). The second through 8^th^ bases, however, showed base preferences.

### Application of MCRE to recombinant protein production

**Figure 3:**
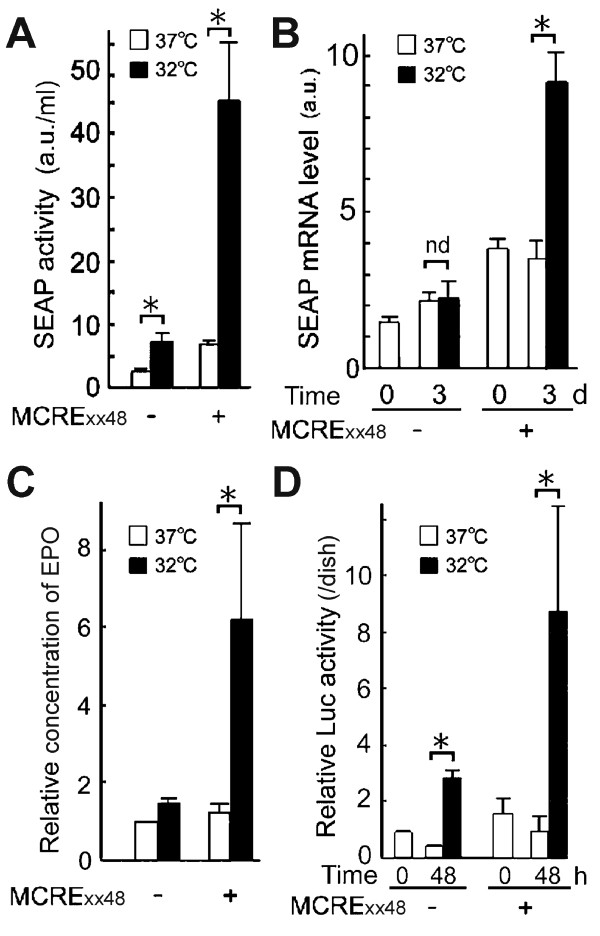
**Enhancement of recombinant protein production at 32°C by MCRE.** (**A**, **B**) CHO-K1 Flp-In cells were stably transfected with the pcDNA5/FRT vector expressing secreted alkaline phosphatase (SEAP) under the control of the CMV promoter with (+) or without (−) an enhancer containing 48 copies of MCRE and its active mutant (MCRExx48). Cells were cultured at 32°C or 37°C for 3 days, and the SEAP activity in culture media (**A**) and mRNA levels in cells (**B**) were analyzed. SEAP mRNAs were quantified by quantitative RT-PCR, and expressed after normalization to those of β-actin. a.u., arbitrary unit. Values are mean ± SE. nd, not different. *, significantly different (p <0.05). (**C**) CHO-K1 Flp-In cells were stably transfected with the pcDNA5/FRT vector expressing erythropoietin (EPO) under the control of the CMV promoter with (+) or without (−) the MCRExx48 enhancer. Cells were cultured at 32°C or 37°C for 3 days, and the EPO concentration in culture media was determined. Values are expressed as relative to the value obtained in cells transfected with plasmids having CMV promoter alone (−) and cultured at 37°C. (**D**) U-2 OS Flp-In cells were stably transfected with the indicated pcDNA5/FRT vector expressing firefly luciferase (Luc) under the control of the CMV promoter with (+) or without (−) MCRExx48. Cells were transferred to 32°C or remained at 37°C for indicated times. Cell extracts were assayed for Luc activity and normalized to the protein level. Values are expressed as relative to the value obtained in cells transfected with plasmids having CMV promoter alone (−) at time 0, and mean ± SE. Experiments were repeated twice (**A**, **B**) and three times (**C**, **D**), with similar results.

Various methods have been developed to increase the yield and reduce the production costs of recombinant proteins, and in many studies human secreted alkaline phosphatase (SEAP) is utilized as a model product [15,16]. To see if MCRE can be utilized to enhance the production of recombinant proteins in stably transfected mammalian cells, we directly connected 3 or 7 copies of 5′-TTCCCCGCCG-3′ without spacer and placed them upstream of the CMV promoter in the pcDNA5/FRT vector expressing SEAP. Then, we transfected HEK293 Flp-In cells with the plasmids to generate stable transfectants. In contrast to the transient transfection experiments described above, the fold induction of SEAP expression at 32°C was not different with and without MCRE. When we used 48 copies of the MCRE and its active mutant (5′-TTCCCGCC-3′, Figure 2C) as enhancers in CHO-K1 Flp-In cells, the amount of SEAP recovered was increased 6.6-fold at 32°C compared with that at 37°C (Figure 3A). In the absence of the enhancer the increase was 3.0-fold at 32°C. Consistently, the SEAP mRNA level was 2.6-fold higher at 32°C compared with 37°C in the presence of the enhancer, but the levels were comparable at 32°C and 37°C in its absence (Figure 3B). As the basal level of expression at 37°C was also increased in the presence of the MCRE enhancer, the yield of SEAP obtained by culture at 32°C was 19.5-fold higher compared with the regular 37°C culture without the enhancer (Figure 3A). The MCRE enhancer was also effective in increasing the production of erythropoietin, a biotechnological protein, in CHO-K1 cells (Figure 3C). The enhancer effect was observed in U-2 OS cells as well (Figure 3D).

### Binding of Sp1 to MCRE

**Figure 4:**
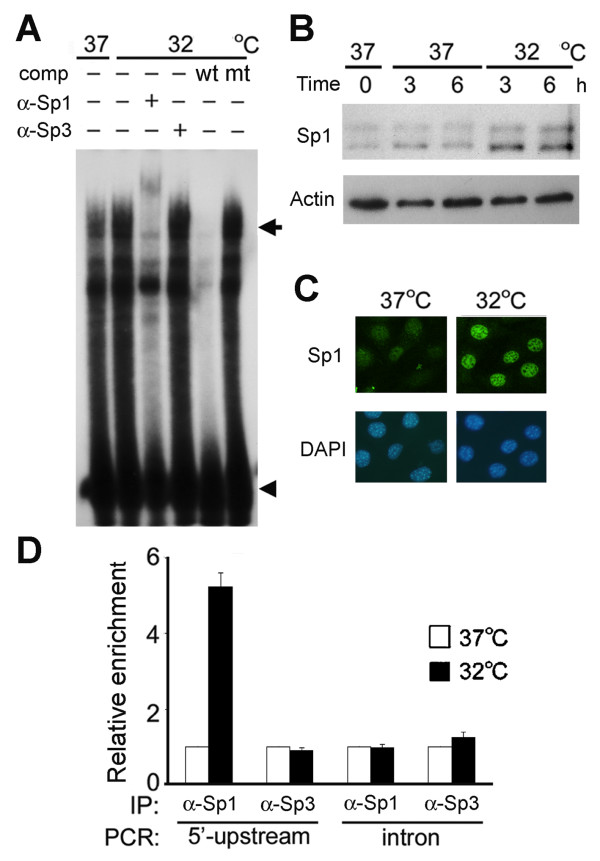
**Recruitment of Sp1 to the *cirp* regulatory region at 32°C.** HeLa cells (**A**) and BALB/3T3 cells (**B**-**D**) were cultured at 37°C, transferred to 32°C or remained at 37°C for 6 hours or indicated times, and then analyzed. (A) EMSA analysis of nuclear extracts using ^32^P-labeled MCRE oligonucleotide as a probe. comp, competition with a 50-fold molar excess of unlabeled wild-type (wt) or mutant (mt) probe. (+) and (−), present and absent, respectively. Arrow, specific probe-protein complex. Arrowhead, free probe. (**B**) Western blotting. Cell lysates were prepared at the indicated times, and analyzed using anti-Sp1 and anti-actin antibodies. (**C**) Immunofluorescence staining of cells. Sp1 was detected with anti-Sp1 antibody and FITC-conjugated anti-mouse IgG, and appears green under the confocal microscope. Nuclei were stained with DAPI, and appear blue. (**D**) ChIP assays. qPCR analysis for the 5′-upstream region containing MCRE and the first intron of the *cirp* gene using chromatins pulled down with anti-Sp1 antibody or anti-Sp3 antibody from cells incubated at the indicated temperatures. The amount of precipitated DNA relative to input (percent of input) was determined for each sample. Values are expressed as relative to those obtained in cells cultured at 37°C. Standard deviations from triplicate PCR reactions are indicated. Experiments were repeated three (**A**, **B**) or four (**C**, **D**) times, with similar results.

To identify the proteins that bind to MCRE and regulate gene expression, the nucleotide sequence of the mouse *cirp* gene 5′-flanking region [NT_039500] was analyzed by using the Transcription Element Search System (TESS) [17]. Since TESS predicted that Sp1 binds to MCRE, we assessed binding of cellular Sp1 to MCRE by electrophoretic mobility shift assay (EMSA). Nuclear protein extracts from HeLa cells contained proteins that bound to the ^32^P-labelled MCRE probe in vitro (Figure 4A). The intensity of one of the bands was increased when cells were cultured at 32°C compared with those cultured at 37°C, and could be competed out by a 50-fold molar excess of the unlabeled wild-type, but not mutant, probe indicating a specific interaction. The addition of anti-Sp1 antibody produced a supershift of the band, whereas the anti-Sp3 antibody had no effect. Western blot analysis demonstrated that the amount of Sp1 protein was increased (Figure 4B), and immunofluorescence microscopy showed that more Sp1 localized in the nucleus (Figure 4C) when cells were cultured at 32°C.

To study whether Sp1 binds to MCRE in the *cirp* regulatory region in intact cells at 32°C, we performed chromatin immunoprecipitation (ChIP) assays in BALB/3T3 cells. We analyzed the 5′ upstream region containing MCRE and the first intron of the *cirp* gene. We detected about a 5-fold enhancement of Sp1 association with the MCRE region, but not with the intron region, at 32°C (Figure 4D). Although both Sp1 and Sp3 preferentially bind to similar DNA motifs, increased binding of Sp3 was not observed, suggesting that mild hypothermia specifically increases recruitment of Sp1 to the *cirp* regulatory region.

### Contribution of Sp1 to increased Cirp expression at 32°C

**Figure 5:**
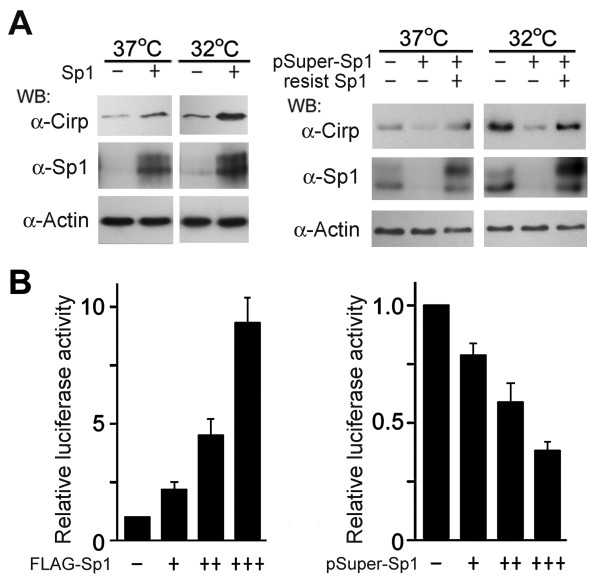
**Contribution of Sp1 to increased expression at 32°C.** (**A**) Effects of Sp1 on endogenous *cirp* expression. U-2 OS (left) and HEK293 (right) cells were transfected with plasmids expressing Sp1 or shRNA for Sp1 (pSuper-Sp1) as indicated, and one day later transferred to 32°C or remained at 37°C. Six hours after the transfer, cell lysates were analyzed by western blotting using anti-Cirp, anti-Sp1 and anti-actin antibodies. In some experiments, plasmids expressing wild-type Sp1 protein from pSuper-Sp1-resistant mRNA (resist Sp1) were also co-transfected. (−), transfection with empty vector. (+), transfection with the indicated plasmids. Experiments were repeated three (left) or four (right) times, with similar results. (**B**) Effects on reporter gene expression. HEK293 cells were co-transfected with the F-Luc reporter plasmids driven by the wild-type −500/+56 *cirp* fragment, pRL-TK, and increasing amounts (+ to +++) of plasmids expressing FLAG-tagged Sp1 (FLAG-Sp1) or pSuper-Sp1 as indicated. One day after transfection, cells were transferred to 32°C or remained at 37°C. Twenty-four hours later, luciferase activities were analyzed. F-Luc activity was normalized to R-Luc activity and expressed as relative to the value obtained in cells co-transfected with the reporter, pRL-TK, and empty vector (−). Values are mean ± SE of triplicates, n = 3.

The binding of more Sp1 to the *cirp* regulatory region does not necessarily imply that Sp1 is involved in the induction of *cirp* expression in response to moderately low temperatures. To confirm this, we first analyzed the effects of Sp1 expression on the endogenous Cirp expression. As shown in Figure 5A, overexpression of Sp1 further increased the Cirp protein levels after transferring the cells to 32°C. Conversely, downregulation of Sp1 by shRNA decreased the Cirp protein levels, and the induction of Cirp at 32°C became less prominent. Rescue of the effects of shRNA by expression of an shRNA-resistant mRNA for wild-type Sp1 indicted that the observed changes were not due to the off-target effects.

The reporter assays further demonstrated that overexpression of Sp1 dose-dependently increased the transcriptional activity of the *cirp* upstream region containing MCRE, whereas downregulation of Sp1 expression suppressed it (Figure 5B).

**Figure 6:**
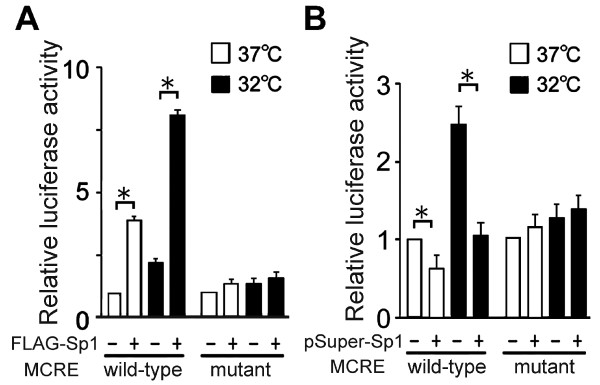
**Effects of MCRE mutation on Sp1 function.** (**A**) HEK293 cells were co-transfected with the F-Luc reporter plasmids driven by the −500/+56 *cirp* fragment containing wild-type or mutant MCRE, pRL-TK, and plasmids expressing FLAG-tagged Sp1 (FLAG-Sp1, A) or shRNA for Sp1 (pSuper-Sp1, **B**) as indicated. One day after transfection, cells were transferred to 32°C or remained at 37°C. Twenty-four hours later, luciferase activities were analyzed. F-Luc activity was normalized to R-Luc activity and expressed as relative to that obtained with wild-type MCRE and without modulation of Sp1 level (−). Values are mean ± SE of triplicates. *, significantly different (p <0.05). Experiments were repeated three times, with similar results.

When MCRE sequences were mutated in the reporter constructs, the enhancement of the reporter gene expression by Sp1 overexpression became minimal (Figure 6A), and the suppressive effects of Sp1 downregulation were not observed (Figure 6B). These results indicate that the effects of Sp1 are mediated by its interaction with the MCRE.

## Discussion

We and others have previously reported that the half-life of *cirp* mRNAs is only marginally influenced by temperature down-shift [2,18]. Consistent with a notion that the transcription rate of *cirp* is increased at moderately low temperatures, we have herein identified a MCRE in the 5′ flanking region of the *cirp* gene that enhances gene expression at 32°C. We further demonstrated that Sp1 binds to MCRE, and that more Sp1 is localized in the nucleus at 32°C to bind to the genomic region containing MCRE. In addition, the reporter assays with mutant MCRE and modulation of the Sp1 expression level showed that the expression of *cirp* is dependent, at least partly, on Sp1 and MCRE and that Sp1 contributes to the cold shock response at 32°C.

*Cis*-regulatory elements like enhancers and promoters are usually characterized by reporter gene assays in cultured cell lines [19]. We did the transient reporter gene assays using HEK293 cells, and identified the MCRE. Although present in two different genomic fragments of *cirp* with enhancer activity, mutagenesis experiments of the identified MCRE octanucleotide revealed that the first base could vary. Furthermore, the binding sequences of Sp1 predicted by TESS in the *cirp* genome did not include the first base of the MCRE, suggesting that the actual element might be the heptanucleotide 5′-CCCCGCC-3′.

The cold-responsive enhancer activity of MCRE was consistently observed when various cell lines including HEK293 and CHO-K1 cells were transiently transfected. In cells stably transfected with similar MCRE constructs, however, the cold-inducible enhancer activity was difficult to observe. This result may not be so surprising because transient reporter gene assays are often a poor proxy for the activities of regulatory elements integrated in the genome, which can be active in narrow windows of development, differentiation and cellular conditions [20]. How the presence of many copies of MCRE seemingly circumvented the problem has yet to be clarified. Although many studies have suggested that recombinant protein production can be increased by culturing at sub-physiological temperatures [12], low temperature also causes growth arrest of cells and suppression of gene expression in general, resulting in variable yields. Thus, specific enhancement of the target gene transcription at 32°C by using MCRE is a promising method to increase the final yield of recombinant proteins in various cell lines. Interestingly, Thaisuchat et al. [21] have recently identified a novel temperature sensitive promoter (222 bp long) in the *S100a6* (calcyclin) gene, which comprises two Sp1 sites.

Sp1 binds GC-rich motifs with high affinity and can regulate the expression of TATA-containing and TATA-less genes via multiple mechanisms [10,11,22]. Sp1 generally activates gene transcription, whereas Sp3 has both transcriptional repressor and activating properties. Discher et al. [23] showed by transient transfection that hypoxia enhances the expression of a reporter gene directed by the pyruvate kinase M promoter in myocytes. The hypoxia response was localized to a conserved GC-rich element that bound Sp1 and Sp3. Hypoxia induced gene expression because it caused depletion of Sp3, removing its transcriptional repression, whereas Sp1 levels remained unchanged. This is unlikely the case with the *cirp* gene, as the western blotting and ChIP assays demonstrated that more Sp1 bound to the chromatin region containing MCRE at 32°C than 37°C, whereas the level of chromatin-bound Sp3 did not differ between these temperatures.

Although Sp1 was once thought to serve mainly as a constitutive activator of housekeeping genes, growing evidence indicates that posttranslational modifications such as phosphorylation, acetylation, sumoylation, ubiquitylation and glycosylation can influence the transcriptional activity and stability of Sp1 [10,22]. For example, Sp1 is phosphorylated at Ser101 by ataxia telangiectasia mutated kinase in response to DNA damage, and the proportion of chromatin-bound phosphorylated Sp1 rapidly increases [24]. Thus, to clarify the molecular mechanisms underlying the observed increase in the amount of Sp1 in the nucleus at 32°C, modifications of Sp1 should be analyzed. Furthermore, Sp1 can regulate the expression of genes via interactions or interplay with other transcription factors such as Ets-1, c-myc, c-Jun and Egr-1, and/or components of the basal transcriptional machinery, and has been linked to chromatin remodeling through interactions with p300 and histone deacetylases [22]. The factors collaborating or competing with ubiquitously expressed Sp1 should be identified to clarify the underlying mechanism of *cirp* induction at moderately low temperatures.

## Conclusions

A novel enhancer element, MCRE, that promotes gene expression 3- to 7-fold in various cell lines upon a temperature shift to 32°C is described. In combination with moderately low culture temperatures, MCRE can be utilized in transient as well as stable transfection to produce large amounts of proteins in a short period of time. By binding Sp1, MCRE contributes to the induction of *cirp* at 32°C. Elucidating the molecular mechanisms of the cold shock response will help develop novel strategies for the treatment of diseases involving cold shock proteins such as male infertility and cancers, and for the production of a variety of diagnostically and therapeutically useful recombinant proteins.

## Methods

### Cell culture

Cells were cultured in Dulbecco’s modified Eagle’s medium supplemented with 10% fetal bovine serum (HEK293 cells, U-2 OS cells, and HeLa cells) or calf serum (BALB/3T3 cells and NIH/3T3 cells), or α medium supplemented with 10% fetal bovine serum (CHO-K1 cells) at 37°C or 32°C in a 5% CO_2_ humidified atmosphere. To produce isogenic stable cell lines, the Flp-In System (Life Technologies) was used. Cells were first transfected with pFRT/lacZeo to generate the Flp-In host cell lines. The calcium phosphate method was used to transfect cells with plasmid DNA as described [25].

### DNA constructs and reporter gene assays

The mouse *cirp* gene spanning about 10 kb upstream of the transcription start site to 56 bp downstream of it was cloned into pBluescript SK(−) (Stratagene). Fragments from this were subcloned into pCAT-Basic or pCAT promoter vectors (Promega). The −500/+56 *cirp* genomic fragment containing mutant MCRE (5′-TCCAAGCC-3′) was generated with the Quick-Change site-directed mutagenesis kit (Stratagene). FLAG-tagged Sp1 was expressed by cloning the coding sequence of Sp1 cDNA ([NM_013672] for mouse and [NM_138473] for human) into the pCMV-3Tag-1 vector (Agilent Technologies). Human Sp1 cDNA was also cloned into the pcDNA3 vector (Invitrogen) and expressed without a tag. For Sp1 protein knockdown, pSuper-Sp1 plasmid was engineered as described [25], with the Sp1 target sequence: 5′-CCTGCAGCAGAATTGAGTC-3′ for mouse, and 5′-GAATCGCACA GTCTCTGGT-3′ for human. To express wild-type Sp1 protein from mRNA resistant to pSuper-Sp1, the human Sp1 cDNA coding sequence was silently mutated at the target sequence to 5′-GAATAGAACA GTCAGTGGT-3′ (mutations underlined), and cloned into the pCMV-3Tag-1 vector.

One day after co-transfection with the pCAT-Basic or pCAT promoter vector-based constructs and CDM8-LacZ, the cells were divided into two groups. One is transferred to 32°C and the other remained at 37°C. Thirty six hours later, cell lysates were prepared and the amount of chloramphenicol acetyltransferase (CAT) protein and β-galactosidase activity were determined using CAT ELISA kit (Roche Diagnostic) and β-galactosidase assay kit (Stratagene), respectively.

To assess the enhancer activity in stably transfected cells, the DNA fragment of interest was placed upstream of the CMV promoter in the pcDNA5/FRT vector (Life Technologies) expressing SEAP or human erythropoietin [NM_000799]. In some experiments, the DNA fragment containing 48 copies of the sequence 5′-TTCCCCGCCGCGTTTCCCGCCG-3′ flanked by *Not*I and *Xba*I recognition sequences (MCRExx48) was used as an enhancer. Flp-In host cell lines were co-transfected with the constructed plasmids and pOG44 (Life Technologies), and the stable transfectants were selected with hygromycin. SEAP activities were assayed as described [26]. Erythropoietin concentrations in cell culture supernatant were measured with the EPO ELISA kit (Roche Diganostics Japan).

For luciferase reporter assays, the firefly luciferase (F-Luc) reporter plasmids driven by the −500/+56 *cirp* genomic fragment containing wild-type or mutant MCRE were constructed and used with the Sp1 expression plasmids, pSuper-Sp1 plasmids or the corresponding empty vectors. The pRL-TK (Promega), carrying the *Renilla* luciferase (R-Luc) under the control of the thymidine kinase promoter, was also used in co-transfection. Cultures were continued at 37°C for 24 hours after transfection, and then transferred to 32°C or remained at 37°C. Twenty-four hours later, cells were harvested and luciferase activities were analyzed using the Dual-Luciferase assay kit (Promega).

Experiments were repeated two to four times, and results were assessed for statistical significance by Student’s t test using the JMP statistical software package (SAS Institute Inc.).

### ChIP assay

ChIP assay was performed using the ChIP-IT Express Enzymatic kit (Active Motif) following the manufacturer’s instructions. Briefly, cells were fixed with formaldehyde, lysed, and the nuclei were incubated in Enzymatic Shearing Cocktail. For each immunoprecipitation, 10 μg of sheared chromatin were incubated overnight at 4°C with 2 μg of normal rabbit IgG, or the corresponding antibodies against Sp1 (sc-14027, Santa Cruz), Sp3 (sc-10252, Santa Cruz), RNA polymerase II (sc-899, Santa Cruz), or normal rabbit IgG (Santa Cruz) and protein G magnetic beads. Anti-RNA polymerase II antibody and IgG were used as positive and negative controls, respectively. Following immunoprecipitation, the cross-linking was reversed by incubation in 0.1 M NaCl for 2 hours at 65°C followed by proteinase-K treatment for 1 hour at 37°C. qPCR was used to determine the amount of precipitated DNA relative to input as (Amount of ChIP DNA)/(Amount of input DNA), and the results were expressed as relative to the value at 37°C. qPCR was performed using ABI-PRISM 7000 (Applied Biosystems) and the SYBR Premix Ex Taq Kit (Takara Bio) with primers for *cirp* 5′ upstream region (−376/-173) (5′-GTCGATGAGTCAAGGTTGGAGCC-3′ and 5′-GTTTTGATTGGCTGGAATCTTTCC-3′) and *cirp* intron (+978/+1153)(5′-CAGGTGTAATCAAGACCTAGAATC-3′ and 5′-AGTTGGAGGCAAAGGAACAGAATC-3′). This *cirp* intron sequence contains no potential Sp1-binding site as assessed with TESS. PCR conditions: 96°C for 2 minutes; 50 cycles of 96°C for 10 seconds, 57°C for 20 seconds, and 72°C for 31 seconds; 72°C for 10 minutes; followed by a post-PCR dissociation curve analysis. Input represents the 0.1% of total pre-immunoprecipitated chromatin. We analyzed 4 independent ChIPs, each in triplicate qPCRs.

### Analyses of gene expression

For RT-qPCR analysis, total RNA was isolated and first-strand cDNA was prepared as described previously [25]. qPCR was performed with primers for SEAP (5′-CTCCAACATGGACATTGACG-3′ and 5′-CCCACCTTGGCTGTAGTCAT-3′), β-actin (5′-AGAGGGAAATTGTGCGTGAC-3′ and 5′-TCTCCAGGGAGGAAGAGGAT-3), F-Luc (5′-TGTGGACGAAGTACCGAAAGGT-3′ and 5′-CCTTCTTGGCCTTTATGAGGATCT-3′), and tubulin, beta3 class III (Tubb3) (5′-CTTTTCGTCTCTAGCCGCGT-3′ and 5′-CTCATCGCTGATGACCTCCC-3). Tubb3 mRNA is known to have a long half-life [27]. The real-time PCRs were set up according to the manufacturer’s instructions (SYBR Premix Ex Taq Kit, Takara Bio). PCR conditions: 95°C for 10 seconds; 41 cycles of 95°C for 5 seconds and 60°C for 34 seconds, followed by a post-PCR dissociation curve analysis. To check the size and specificity of the reaction, PCR products were run on agarose gels, stained with ethidium bromide, and photographed under UV light. In some experiments, PCR products were extracted from the gels, cloned by TA cloning, and their DNA sequences were verified.

Western blotting and immunofluorescence microscopy were performed as described [2,9] using anti-Cirp, anti-FLAG, anti-β actin, anti-Sp1, and FITC-conjugated anti-rabbit immunoglobulin (Dako) antibodies. RNAs were isolated from cells using. ISOGENE (Nippon Gene), and northern blotting was performed as described [2].

### Nuclear extracts and EMSA

Extraction of nuclear proteins and EMSA were performed essentially as described [25]. ^32^P-labeled double stranded oligonucleotide (5′-TCAGCTGTCCCCGCCTCCTAGT-3′) was used as a probe. For competition and supershift experiments, proteins were preincubated with the unlabeled probe, its mutant (5′- TCAGCTGTCCAAGCCTCCTAGT-3′), the anti-Sp1 antibody or anti-Sp3 antibody for 1 hour at 4°C. Labeled probes were then added, and the reaction tubes were incubated for 30 min at room temperature.

## Abbreviations

CAT: Chloramphenicol acetyltransferase; ChIP: Chromatin immunoprecipitation; Cirp: Cold-inducible RNA-binding protein; EMSA: Electrophoretic mobility shift assay; F-Luc: Firefly luciferase; MCRE: Mild-cold responsive element; Rbm3: RNA binding motif protein 3; R-Luc: *Renilla* luciferase; SEAP: Secreted alkaline phosphatase; Sp1: Specificity protein 1; TESS: Transcription Element Search System.

## Competing interests

The authors declare that they have no competing interests.

## Authors’ contributions

YS carried out the molecular genetic studies and drafted the manuscript. HirH participated in the design of the study and performed the molecular analysis. HisH carried out the molecular and cellular biological studies.YL carried out the molecular genetic studies. TF participated in the design of the study and performed the statistical analysis. TS carried out the molecular biological studies. MMC carried out the molecular biological studies and helped to draft the manuscript. KI participated in the design of the probes and sequence analysis. TC participated in study design and coordination. JF conceived of the study, carried out the reporter gene assays, and participated in its design and coordination and helped to draft the manuscript. All authors read and approved the final manuscript.
